# Parasites of poultry in Nigeria from 1980 to 2022: a review

**DOI:** 10.1007/s12639-025-01792-5

**Published:** 2025-03-06

**Authors:** Chahari A. Midala, Falmata Kyari, Oriel Thekisoe, ThankGod E. Onyiche

**Affiliations:** 1 Department of Veterinary Parasitology and Entomology, Faculty of Veterinary Medicine, University of Maiduguri, Maiduguri, Nigeria; 2 Unit for Environmental Sciences and Management, North-West University, Potchefstroom, 2531 South Africa

**Keywords:** Internal parasites, Helminths, Protozoa, Blood parasites, External parasites, Nigeria

## Abstract

Poultry production is crucial for food and nutrition security level through the provision of eggs and meat and it also generates income. However, parasitic diseases are among the major constraints to the poultry industry across the world as they can affect the health, welfare, and production performance. These parasitic diseases can be broadly classified as endo-, ecto- and haemoparasites and they occur as single infection or in combination and affect productivity of poultry in Nigeria. This review focuses on summarizing all the major classes of parasitic diseases of poultry in Nigeria from 1980 to 2022. We search two electronic databases (Google Scholar and AJOL) to retrieve relevant articles published from 1980 to 2022 across the six geopolitical zones of Nigeria comprising both North and South. Our findings regarding endo-parasites, indicates that the major species of nematodes registered includes *Ascaridia galli*, *Heterakis gallinarum*, *Subuluru brumpti* and *Capillaria* species. As per cestodes, some of the documented species from published literature were *Raillietina* species (specifically *R. tetragona*, *R. cesticillus* and *R. echinobothrida*) and *Choanotaenia infundibulum*. *Prosthogonimus species* was the only documented trematodes infecting poultry in Nigeria. On the side of ecto-parasites, almost all the major groups which include ticks (*Argas persicus*, and *Ornothodoros moubata*), mites (*Dermanyssus gallinae* and *Knemidocoptes mutans*), fleas (*Echinophaga gallinacean*) and lice (*Menacanthus stramineus*,* Menopon gallinae*, *Lipeurus caponis* and *Columbicola columbae*) have all been documented infesting poultry across the country. Lastly, protozoan parasites including haemoparasites (*Plasmodium* spp., *Haemoproteus* spp. and *Leucocytozoon* spp.) and coccidian (*Eimeria* spp. and *Cryptosporidium* spp.) were observed to infect poultry in Nigeria. In conclusion, this review has provided available information on the occurrence and distribution of the ecto-, endo- and haemoparasites in different types of poultry in Nigeria.

## Introduction

The provision of meat and eggs to meet the animal-based protein requirement across several communities worldwide is regarded as one of the most important contribution of the poultry industry. The Food and Agricultural Organization (FAO) describe poultry farming as a significant contributor to food, nutrition, and financial security throughout the world (FAO 2000). Of all meat types consumed in Africa, poultry meat contributes approximately 25% while as in some parts of the continent; it represents about 100% of the available animal protein (Idowu et al. 2019).

Chickens (including indigenous chickens, layers, broilers and cockerels), turkeys, ducks, guineafowls, pigeons, pheasant, quails, and ostriches are specific birds that makes up domestic poultry production (Idowu et al. 2019; Mohammed and Obeta 2015; Lawal et al. 2016a, b). However, chickens and turkeys contribute the most in commercial poultry farming (Opara et al. 2012, 2014; Lawal et al. 2016a, b).

In Nigeria, poultry meat and eggs are the most widely consumed animal proteins, regardless of religious or cultural affiliation (USDA 2013), this plays an essential part in the provision of animal protein to humans, as well as in the development of the national economy as a source of revenue (FAOSTAT 2005), with backyard poultry accounting for more than 60% of total national poultry flocks in most African nations, including Nigeria, with an estimated asset value of more than £3.98 billion (₦1.734 trillion) (Getu 2014; Nghonjuyi et al. 2014; Mohammed and Obeta 2015). It is estimated that the poultry population in Nigeria totals 160 million birds, with chickens accounting for approximately 72.4 million of that number and backyard poultry production accounts for around 43.4 million of this total, making it the most important type of chicken production (Akintunde et al. 2015; Mohammed and Obeta 2015). This industry contributes up to 15% to the country’s gross domestic product (GDP), which accounts for 36% of the country’s total protein intake (Akintunde et al. 2015), making it an important component of the livestock subsector, which has progressed to the level of commercial operation with thousands of birds that are used to provide employment, income, and animal protein to both urban and rural dwellers in Nigeria, as well as manure for crop production (Balami et al. 2014). In most developing nations, obtaining optimum poultry production has been impeded by a variety of problems, among them are parasitic diseases affecting poultry flocks (Mapiye et al. 2008) which, have continued to be a serious obstacle to the expansion and profitability in developing agricultural countries (Mohammed and Obeta 2015).

Parasitism in poultry production consist of those parasites that are found within gastrointestinal tract and are termed as ‘endoparasites’ while those found outside the gastrointestinal tract, most often infesting the skin and soft tissues are termed “ectoparasites’. Gastrointestinal helminthiasis could be caused by tapeworms (cestodes), flukes (trematodes) and roundworms (nematodes) (Macklin 2013; Ola-Fadunsin et al. 2019a), with nematodes been the most important intestinal worms of poultry (Bachaya et al. 2015; Ola-Fadunsin et al. 2019b). On the other hand, mites, lice, ticks, and fleas are common poultry ectoparasites (Saidu et al. 1994), with lice being the most frequently reported among chickens (Benbrook 1965). Parasitism results in significant production losses due to stunted growth, decreased egg production, emaciation, anemia, and mortality (Kaufman et al. 2007). It has also been reported that, parasitic infections or their concomitant infections have been documented to cause immunosuppression and a poor immune response to immunizations against certain poultry diseases (Anna 2006).

Available evidence suggests that many researchers have documented studies on specific endoparasites and ectoparasites affecting poultry across the geopolitical zones in Nigeria. Nationwide status and burden of endo- and ecto-parasitic species affecting poultry is yet to be documented and consolidated into a single one-stop reference point for future studies. Consequently, this study attempts to fill up knowledge gaps concerning the distribution and impact of parasitic diseases of poultry in Nigeria hitherto those species that are poorly understood. We are of the opinion that the understanding of parasitic species affecting birds will help in devising measures to improve health and productivity of these birds (Msoffe et al. 2010). Hence this review seeks to address gap in knowledge by collating and analyzing data from literatures regarding endo- and ecto-parasites of poultry in Nigeria.

## Materials and methods

### Data collection and extraction

Electronic search of key databases (Google Scholar, and AJOL) was conducted in February, 2022 to collect relevant published articles with no limitation on the publication year. The following combination of terms was used for the search string: (Parasites OR Ectoparasites OR Endoparasite or Haemoparasites) and (Avain OR Birds OR Poultry OR Chickens) and Nigeria. The inclusion criteria adopted in this study includes the following (i) cross sectional study involving the prevalence of any parasite type of poultry and other avian species in Nigeria (ii) study written in English language and published from 1980 to 2022, (iii) the full-text of the article is available (iv) the diagnostic technique employed in the determination of the parasite was clearly capture in the methodology (v) the number of animals screened for the parasite type and the number of positives was clearly captured. A suitable data extraction template was prepared to collect and organize information from each reviewed study using excel. The information retrieved included author, publication year, parasite species encountered, avian host, geographical location, sample size, prevalence and predilection sites.

## Results

### Search outcome

The articles retrieved following search was downloaded using the title of the article that captures any locality/region within Nigeria and investigated the prevalence and abundance of any parasite type of poultry. The files were organized into folders according to the class of parasites identified as either endo-, ecto- and haemoparasites. When several parasites types were reported, all those articles were placed together in one folder.

An overview of the different parasite types is synthesized and presented below. For ectoparasites, we reported below the different class of ectoparasites encountered so far in poultry including fleas, lice, tick and mites whereas for endoparasites we recorded cestodes, nematodes and trematodes. Lastly for haemoparasites, we registered *Plasmodium*, *Haemoproteus* and *Leucocytozoon* species.

### Ectoparasite

**Fig. 1:**
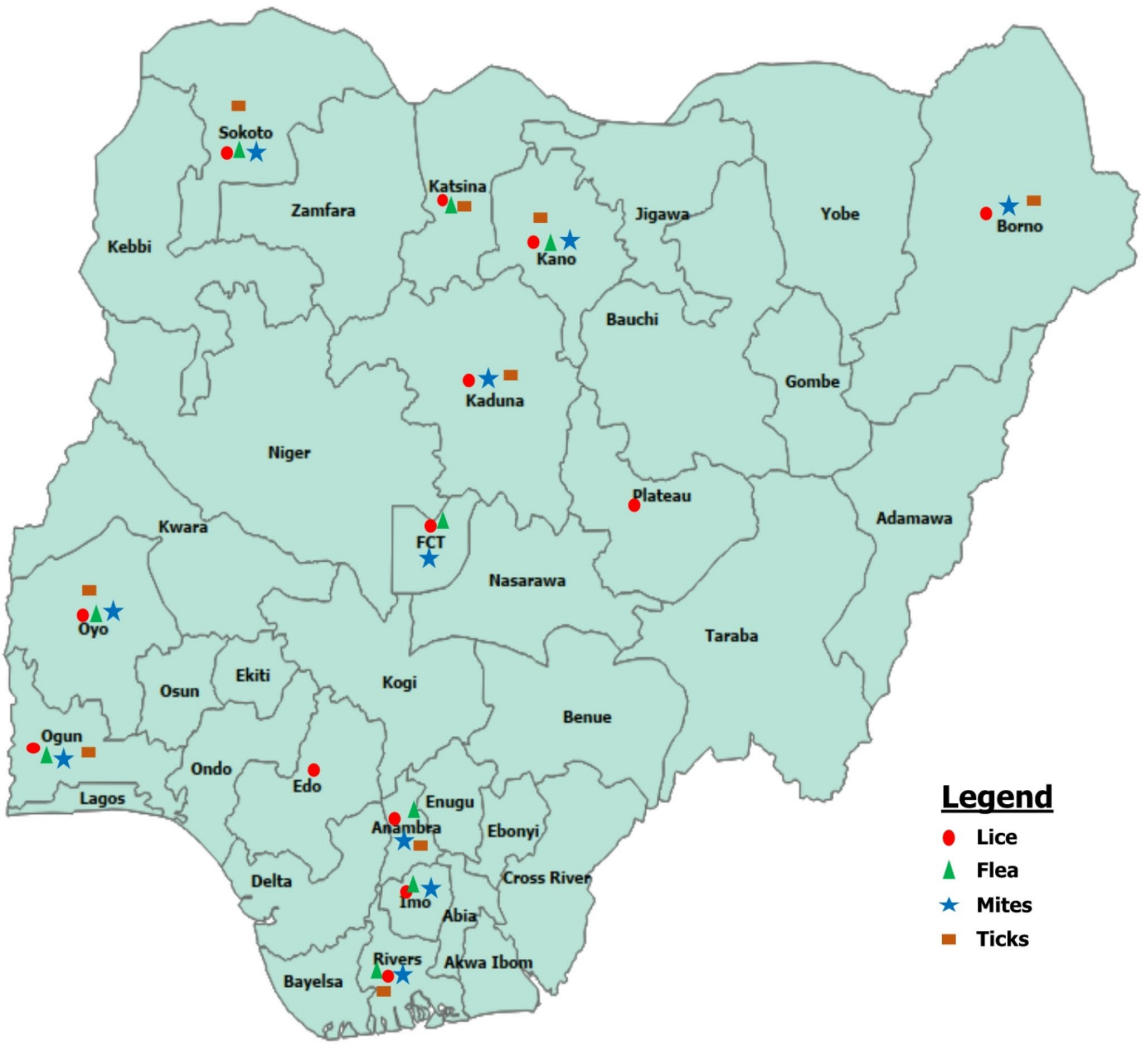
Geographical distribution of Ectoparasites of birds across Nigeria

Ectoparasites of birds belong to the phylum *Arthropoda*, made up of two classes: *Arachnida* containing the order *Acarina* (ticks and mites) and *Insecta* with the orders *Phthiraptera* (lice), and *Siphonaprera* (fleas) (Permin and Hansen 1998). These arthropods are responsible for bad health and poor growth and condition of birds (Endale et al. 2023). Their activities directly cause itchiness, tissue damage, pain, allergies, toxicosis, bleeding and dermatitis which ultimately affect the output and quality of meat and eggs, and indirectly, they serve as biological or mechanical vectors for the transmission of a variety of infections (Boseret et al. 2013; Rezaei et al. 2016; Tessema 2019). For instance, ticks and mites are the carriers of various diseases that affect poultry (Rezaei et al. 2016a). Additionally, *Dermanyssus gallinae* has been reported to transmit animal and human pathogens including viruses, bacteria, and parasites (Ebani and Mancianti 2021). Diverse array of arthropods constitutes the major ectoparasites of poultry such as fleas, bugs, lice, mites, and ticks (Endale et al. 2023). The production method influences the degree and types of infestation, as they live on or in the skin and feathers of their host (Endale et al. 2023). They are most common in chickens raised traditionally in backyard flocks. Most ectoparasites (lice, for example) are host specific, whereas many other ectoparasites (for example, ticks and mites) travel from one host to another on a regular basis (Yacob et al. 2009). Typically, ectoparasite feeds on their host’s blood, feathers, skin, and scales, this is accompanied by a wide range of symptoms such as loss of feathers, stunted growth, poor egg production and hatchability, anemia, increased mortality as well as susceptibility to other infections (Urquhart et al. 1996; Mirzaei et al. 2016). One essential determinant of ecto-parasite infestation in chickens in Nigeria is climatic condition, with high prevalence recorded during periods of high humidity especially during the rainy season (Lawal et al. 2017). Fluctuations in temperature and humidity in different seasons of the year determine the survival and perpetuation of ecto-parasites with subsequent increase in bird infestations (Belete et al. 2016). The geographical distribution of ecto-parasites across Nigeria is represented in Fig. 1. Most often poor management and lack of parasite control programs is associated with the infestation of chickens by different species of lice, flea, ticks and mites (Mungube et al. 2008).

#### Fleas

Fleas belong to the order *Siphonaptera* and are small, wing-less, obligate, blood-feeding insects.

Most fleas’ craw through their hosts feathers but are rarely detected there as they spend majority of their time in the host’s nest. They only climb out to feed or disperse (Hopla 1994). The bites from fleas are annoying leading to discomfort, skin allergy and secondary infection (Wall and Shearer 2001; Dieme et al. 2015). Furthermore, flea infestations cause skin problems (dermatitis), pruritus and intense itching in affected hosts (Koutinas et al. 1995). Several researchers have reported flea infestation in poultry species across all the geopolitical zones of Nigeria except for Northeast. Citations for the different geopolitical zones includes North-west (Jamilu et al. 2018; Ahaotu et al. 2019; Bala et al. 2011), North-central (Odenu et al. 2016), South-east (Onyekachi 2021a, b; Ahaotu et al. 2019; Ehisianya et al. 2021; Ikpeze et al. 2008), South-south (Ahaotu et al. 2019) and South-west (Ahaotu et al. 2019). The Hen flea or sticktight flea, *Echinophaga gallinacean* is the only species of flea reported to infest birds in Nigeria with the flea been reported around the head, wattle, eye lid, and comb (Odenu et al. 2016; Ahaotu et al. 2029; Ikpeze et al. 2008), while Ehisianya et al. (2021) report the presence of the flea around the wings and under feather with a prevalence of 5.5% in chicken, guinea fowl, and pigeon. Other researchers have reported the occurrence of this flea with vary prevalence such as 29.1% in chicken (Onyekachi 2021a, b), 5.9% in local chicken (Odenu et al. 2016), 1.06% in domestic chicken (Jamilu et al. 2018), 27.3% in indigenous chicken (Ahaotu et al. 2019), 10.6% (Bala et al. 2011), and 69.37% among chicken (Ikpeze et al. 2008). An overview of the documentary evidence of *Echinophaga gallinacean* across Nigeria is presented in Table 1.

**Table 1:** Distribution and prevalence of *Echinophaga gallinacean* in poultry across the geopolitical zones in Nigeria

Geographical region	Location	Avian type	Reported predilection site	Number examined	No. of positive samples	Prevalence (%)	References
South-East	Anambra	Chicken	-	300	42	29.10	Onyekachi (2021a, b)
North-Central	Abuja	Local chickens	Head; eye, comb, wattle	250	12	5.90	Odenu et al. (2016)
North-West	Katsina	Domestic chicken	-	944	10	1.06	Jamilu et al. (2018)
North-West, South-East, South-South, South-West	Anambra, Ogun, Anambra, Sokoto, Rivers, Oyo, Kano, Imo	Domestic chickens	Comb, wattles, eyes, ears	1025	280	27.30	Ahaotu et al. (2019)
North-West	Sokoto	Domestic chicken	-	160	17	10.60	Bala et al. (2011)
South-East	Imo	Poultry (chicken, guinea fowl, pigeon) Ostrich	Wing, under feather	200	9	5.45	Ehisianya et al. (2021)
South-East	Anambra	Chicken	Comb, wattles, eye lid	4650	3087	69.37	Ikpeze et al. (2008)

#### Lice

Lice are wingless haematophagous ectoparasites that commonly parasitize animals and birds (Ikpeze 2008), and they can reproduce quickly and cause large-scale infestations (Ruff 1999). The blood-feeding behaviours and chewing of lice results in anaemia, skin irritation, decreased weight gain, low egg production and reduce intake of feed with negative consequence to the performance of the flock (Wall and Shearer 2001). Lice have been reported to have the highest prevalence than any other ectoparasites by most researchers across all the geopolitical zones in Nigeria (Odenu et al. 2016; Jamilu et al. 2018; Zaria et al. 1996; Lawal et al. 2019; Bala et al. 2011; Ehisianya et al. 2021; Ekpo et al. 2010; Adamu et al. 2015; Edosomwan and Igetei 2018; Ahaotu et al. 2019; Ikpeze et al. 2008; Fabiyi 1980; Adang et al. 2008, 2009a, b). These wide prevalence highlights their ability to adapt favorably in both humid and hot conditions. Available records in Nigeria show that about four different types of lice have been documented in birds including body lice (*Menacanthus stramineus*), feather shaft lice *(Menopon gallinae*), wing or feather lice (*Lipeurus caponis*) and the pigeon louse (*Columbicola columbae*).

*Menacanthus stramineus* is the most frequently reported lice species affecting poultry in Nigeria, with Odenu et al. (2016) reporting a prevalence of 61.4% and 89.5% all over the body of local chicken and exotic birds, respectively. A prevalence of 90% has been documented on the skin surface of free-range chickens (Ekpo et al. 2010), Ehisianya et al. (2021) reported a prevalence of 84.24% around the neck, trunk, wings, and under feathers of poultry birds. However, lower prevalences have also been reported, with 0.85% in domestic chickens (Jamilu et al. 2018); 6.9% in chickens (Bala et al. 2011), 8.33% in turkeys (Lawal et al. 2019) while Adamu et al. (2015) reported a prevalence of 2% of *Menacanthus spp.* infestation in Japanese quails; 5.5% in chicken, pigeon, and ducks (Edosomwan and Igetei 2018). Secondly, the wing or feather lice *Lipeurus caponis* have also been documented in chickens in Nigeria (Odenu et al. 2016; Ekpo et al. 2010; Edosomwan and Igetei 2018; Ahaotu et al. 2019; Ikpeze et al. 2008; Fabiyi 1980). Low prevalence of 2.0% has been documented in Japanaese quails in Sokoto State (Adamu et al. 2015). Furthermore, the poultry shaft louse *Menopon gallinae* was predominately reported in the North-West geopolitical zone (Bala et al. 2011; Ahaotu et al. 2019; Adang et al. 2008, 2009a, b). Prevalences of 50.0% (Ahaotu et al. 2019), 31.90% (Ikpeze et al. 2008) and 8.10% (Bala et al. 2011) was reported in chicken, while Adang et al. (2008) reported a prevalence of 8.40% in Laughing doves and a prevalence of 2.50% and 20% was reported in Black-billed wood doves and Vinaceous doves respectively (Adang et al. 2009a). Lastly, the pigeon louse *Columbicola columbae* were mainly reported from Zaria, Kaduna State in North-West Nigeria (Adang et al. 2008, 2009a, b). Edosomwan and Igetei (2018) reported a prevalence of 7.78% in chicken, pigeon and ducks, from Edo (South-South). They are commonly seen around the tail and quill feather of the wings. Tables 2, 3, 4 and 5 showcase the distribution and prevalence of *Menacanthus stramineus*, *Lipeurus caponis*,* Menopon gallinae* and *Columbicola columbae* respectively from birds across Nigeria.

**Table 2:** Distribution and prevalence of *Menacanthus stramineus* in poultry across the geopolitical zones in Nigeria

Geographical region	Location	Avian type	Reported predilection site	Number examined	No. of positive samples	Prevalence (%)	References
North-Central	Abuja	Local chicken	All over the body	250	124	61.40	Odenu et al. (2016)
		Exotic birds		50	17	89.50	
North-West	Katsina	Domestic chicken	-	944	8	0.85	Jamilu et al. (2018)
North-East	Borno	Domestic fowl	-	663	324	42.46	Zaria et al. (1996)
North-East	Borno	Turkey	-	300	25	8.33	Lawal et al. (2019)
North-West	Sokoto	Chicken	-	160	11	6.90	Bala et al. (2011)
South-East	Owerri	Poultry	Neck, trunk, wing, under feather, thigh, body and perineum	200	139	84.24	Ehisianya et al. (2021)
South-West	Abeokuta	Free range chicken	Skin	20	18	90.00	Ekpo et al. (2010)
North-West	Sokoto	Japanese Quails	-	200	4	2.00	Adamu et al. (2015)
South-South	Edo	Chicken, pigeon, ducks	-	23	5	5.50	Edosomwan and Igetei (2018)

**Table 3:** Distribution and prevalence of *Lipeurus caponis* in poultry across the geopolitical zones in Nigeria

Geographical region	Location	Avian type	Reported predilection site	Number examined	No. of positive samples	Prevalence (%)	References
North-Central	Abuja	Local chicken	Under the ventral abdomen	250	18	8.90	Odenu et al. (2016)
		Exotic birds		27	6	22.20	
North-West, South-East, South-South, South-West	Akwa, Ogun, Anambra, Sokoto, Rivers, Oyo, Kano, Imo	Domestic chicken	Wing and feathers	1025	227	22.10	Ahaotu et al. (2019)
North-West	Sokoto	Chickens	-	160	8	5.00	Bala et al. (2011)
North-West	Sokoto	Japanese Quails	-	200	4	2.00	Adamu et al. (2015)
South-East	Anambra	Chickens	Wings, body surface and shafts	4650	1935	41.61	Ikpeze et al. (2008)
North-Central	Plateau	Chickens	-	400	192	48.00	Fabiyi (1980)
South-West	Ogun	Free range chickens	Feathers	20	12	60.00	Ekpo et al. (2010)
South-South	Edo	Chicken, pigeon, ducks	-	23	17	18.89	Edosomwan and Igetei (2018)

**Table 4:** Distribution and prevalence of *Menopon gallinae* in poultry across the geopolitical zones in Nigeria

Geographical region	Location	Avian type	Reported predilection site	Number examined	No. of positive samples	Prevalence (%)	References
North-West, South-East, South-South, South-West	Anambra, Ogun, Anambra, Sokoto, Rivers, Oyo, Kano, Imo	Domestic chickens	Feather, shafts, all over the body	1025	513	50.00	Ahaotu et al. (2019)
North-West	Sokoto	Chickens	-	160	13	8.10	Bala et al. (2011)
North-West	Kaduna	Laughing doves	Body, head, neck	382	32	8.40	Adang et al. (2008)
South-East	Anambra	Chickens	wings, body surface, shafts	4650	2205	31.90	Ikpeze et al. (2008)
North-West	Kaduna	Black-billed wood doves	Body, head, neck	40	1	2.50	Adang et al. (2009a)
		Vinaceous doves		10	2	20.00	
North-West	Kaduna	Pigeon	Body, head, neck	30	17	56.70	Adang et al. (2009b)
South-South	Edo	Chicken, pigeon, ducks	-	23	21	23.33	Edosomwan and Igetei (2018)

**Table 5:** Distribution and prevalence of *Columbicola columbae* in poultry across the geopolitical zones in Nigeria

Geographical region	Location	Avian type	Reported predilection site	Number examined	No. of positive samples	Prevalence (%)	References
North-West	Kaduna	Laughing doves	Quill feather, wings, tail	382	37	9.70	Adang et al. (2008)
North-West	Kaduna	Black-billed wood doves	Quill feathers of wings and tail	40	5	12.50	Adang et al. (2009a)
		Vinaceous doves		10	1	10.00	
North-West	Kaduna	Pigeon	Quill feather and tail	30	18	60.00	Adang et al. (2009b)
South-South	Edo	Chicken, pigeon, ducks	-	23	7	7.78	Edosomwan and Igetei (2018)

#### Ticks

Ticks are obligatory haematophagous ectoparasitic organism for portions or the entirety of their life cycle (Hoplea et al. 1994). They are frequently seen clinging to the birds’ naked skin, such as under wings, feet, brood patches, eyelids, and heads, where they feed by sucking blood (Arends 2003). Ticks act as hosts for a wide variety of zoonotic infections, and in recent years, researchers have focused on the relative significance of birds in maintaining tick life cycles and serving as reservoirs for vector-borne pathogens (Brinkerhoff et al. 2011; Hasle 2013; Loss 2016). In addition to the potentially significant importance in the spread of tick-borne diseases across new geographic areas, it is possible that birds could also play a role in the enzootic maintenance of tick-borne pathogens (Hamer et al. 2011; Scott et al. 2012). Ticks infestation can be controlled by the use of approved acaricide. It is also important to build chicken houses without cracks or crevices where ticks can hide (Wade 2010).

*Argas persicus* is predominant tick specie reported by researchers, mostly from the north-west geopolitical zone (Jamilu et al. 2018; Ahaotu et al. 2019; Bala et al. 2011; Bunza et al. 2008; Adang et al. 2008, 2009a). Onyekachi (2021a, b) reported a prevalence of 12.5% from chicken in Akwa, Anambra State in south-eastern Nigeria. Also, a prevalence of 31.72% was documented in domestic fowl (Zaria et al. 1996), and 2.67% in turkey (Lawal et al. 2019) from Maiduguri and Jere Local Government Areas in Borno State, North-East, Nigeria. These ticks were predominately found underneath the wings and breast area (Ahaotu et al. 2019; Bunza et al. 2008) and around the wing web and thigh (Adang et al. 2008, 2009a). An overview of the distribution and prevalence of *A. persicus* in Nigeria is presented in Table 6. Other species of ticks so far documented includes *Argas walkerae* and *Ornothodoros moubata* from chickens and guinea fowl in Sokoto north-western Nigeria (Bunza et al. 2008).

**Table 6:** Distribution and prevalence of *Argas persicus* in poultry across the geopolitical zones in Nigeria

Geographical region	Location	Avian type	Reported predilection site	Number examined	No. of positive samples	Prevalence (%)	References
South-East	Anambra	Chicken	-	300	18	12.50	Onyekachi (2021a, b)
North-West	Katsina	Domestic chickens	-	944	40	4.24	Jamilu et al. (2018)
North-East	Borno	Domestic fowl	-	663	242	31.72	Zaria et al. (1996)
North-East	Borno	Turkey	-	300	8	2.67	Lawal et al. (2019)
North-West, South-East, South-South, South-West	Ogun, Anambra, Sokoto, Rivers, Oyo, Kano, Imo	Domestic chicken	Ventral abdomen beneath the wings	1025	64	6.20	Ahaotu et al. (2019)
North-West	Sokoto	Chicken	-	160	14	8.80	Bala et al. (2011)
North-West	Sokoto	Chicken	Under wings, breast area, vents	150	38	62.20	Bunza et al. (2008)
		Guinea fowl		150	16	57.20	
North-West	Kaduna	Laughing doves	Wing web, thigh	382	12	3.10	Adang et al. (2008)
North-West	Kaduna	Black-billed wood doves	Wing web	40	2	5.00	Adang et al. (2009a)

#### Mites

Infestation by mites in poultry cause listlessness, blood loss and scratching results in drop in production and performance with the birds abandoning their brooding nests (Gless and Raun 1959). Other effects include loss of weight and feathers, reduced egg production and even death. The major mite species afflicting poultry are the burrowing (chewing) or hematophagus (blood sucking) mites. The red poultry mite (*Dermanyssus gallinae*), Northern fowl mite (*Ornithonyssus sylviarum*), and Tropical fowl mite (*Ornithonyssus bursa*) are blood sucking mites that live exclusively on poultry with the exception of *Dermanyssus gallinae* which are nocturnal and hide in crevices during the day (Hopla et al. 1994). The scaly leg mite (*Knemidocoptes mutans*) is the most important burrowing mite in poultry, found on the scales of the legs, beak, and occasionally on the comb, wattle, and neck (Williams 2010). Due to the fact that most birds under extensive management are infected with more than one type of parasite, the impact of ectoparasite infestations may be even more detrimental with concurrent parasite infestations, resulting in immunosuppression and high mortality (Hopla, 1994; Nnadi and George 2010).

*Dermanyssus gallinae* was widely reported across all geopolitical zones in Nigeria with the exception of the northcentral zone (Onyekachi 2021a, b; Zaria et al. 1996; Lawal et al. 2019; Ahaotu et al. 2019; Adang et al. 2008). This parasite where seen mostly over the entire body of indigenous chickens (Ahaotu et al. 2019) and around the legs of laughing doves (Adang et al. 2008) with a prevalence of 13.9% and 0.26% respectively. *Knemidocoptes mutans* was reported across all geopolitical zones (Odenu et al. 2016; Zaria et al. 1996; Ahaotu et al. 2019; Bala et al. 2011; Ehisianya et al. 2021; Ikpeze et al. 2008), and were mainly seen around the shanks, lower limb, and feet (Odenu et al. 2016; Ehisianya et al. 2021; Ikpeze et al. 2008; Ahaotu et al. 2019), and non-feathered areas (Ahaotu et al. 2019). Other species of mites reported are *Cnemidocoptes gallinae* reported in chickens with a prevalence of 8.1% (Bala et al. 2011) and *Epidermoptes bilbobatus* in turkeys with a prevalence of 3% (Lawal et al. 2019). An overview of two of the commonly encountered mites of poultry in Nigeria is presented in Tables 7 and 8.

**Table 7:** Distribution and prevalence of *Dermanyssus gallinae* in poultry across the geopolitical zones in Nigeria

Geographical region	Location	Avian type	Reported predilection site	No. examined	No. of positive samples	Prevalence (%)	References
South-East	Anambra	Chicken	-	300	36	25.00	Onyekachi (2021a, b)
North-East	Borno	Domestic fowl	-	663	3	0.39	Zaria et al. (1996)
North-East	Borno	Turkey	-	300	21	7.00	Lawal et al. (2019)
North-West, South-East, South-South, South-West	Ogun, Anambra, Sokoto, Rivers, Oyo, Kano, Imo	Domestic chicken	Entire body	1025	142	13.90	Ahaotu et al. (2019)
North-West	Kaduna	Laughing doves	Legs	382	1	0.26	Adang et al. (2008)

**Table 8:** Distribution and prevalence of *Knemidocoptes mutans* in poultry across the geopolitical zones in Nigeria

Geographical region	Location	Avian type	Reported predilection site	Number examined	No. of positive samples	Prevalence (%)	References
North-Central	Gwagwalada, Abuja	Local chicken	Shanks	250	18	8.9	Odenu et al. (2016)
North-East	Maiduguri, Borno	Domestic fowl	-	663	40	5.24	Zaria et al. (1996)
North-West, South-East, South-South, South-West	Akwa, Ogun, Anambra, Sokoto, Rivers, Oyo, Kano, Imo	Domestic chickens	lower limb; non feathered area	1025	347	33.9	Ahaotu et al. (2019)
North-West	Sokoto	Chickens	-	160	15	9.4	Bala et al. (2011)
South-East	Imo	Poultry; chickens, guinea fowl, pigeon, ostrich	Feet	200	17	10.3	Ehisianya et al. (2021)
South-South	Anambra	Chickens	Feet, leg	4650	1679	27.7	Ikpeze et al. (2008)

### Endoparasites

Single-celled protozoans and worms (helminths) are examples of endo-parasites that can infiltrate nearly all the organs of their hosts’ bodies. It is one of the most common clinical problems in birds, and it is at the top of the list of clinical problems considered for differential diagnosis, particularly in newly acquired birds and in large aviary collections (Philips 2000). It has been reported that gastro-intestinal parasitism is the most common and important parasite found in caged and aviary birds (Greve 1996). Birds on free-range system are exposed to diverse environment, which negatively increase their risk of acquire ova, larva and oocysts of parasites from the soil during the scavenging process with resultant high and diverse endo-parasites infection in birds (Adejinmi and Oke 2011).

**Fig. 2:**
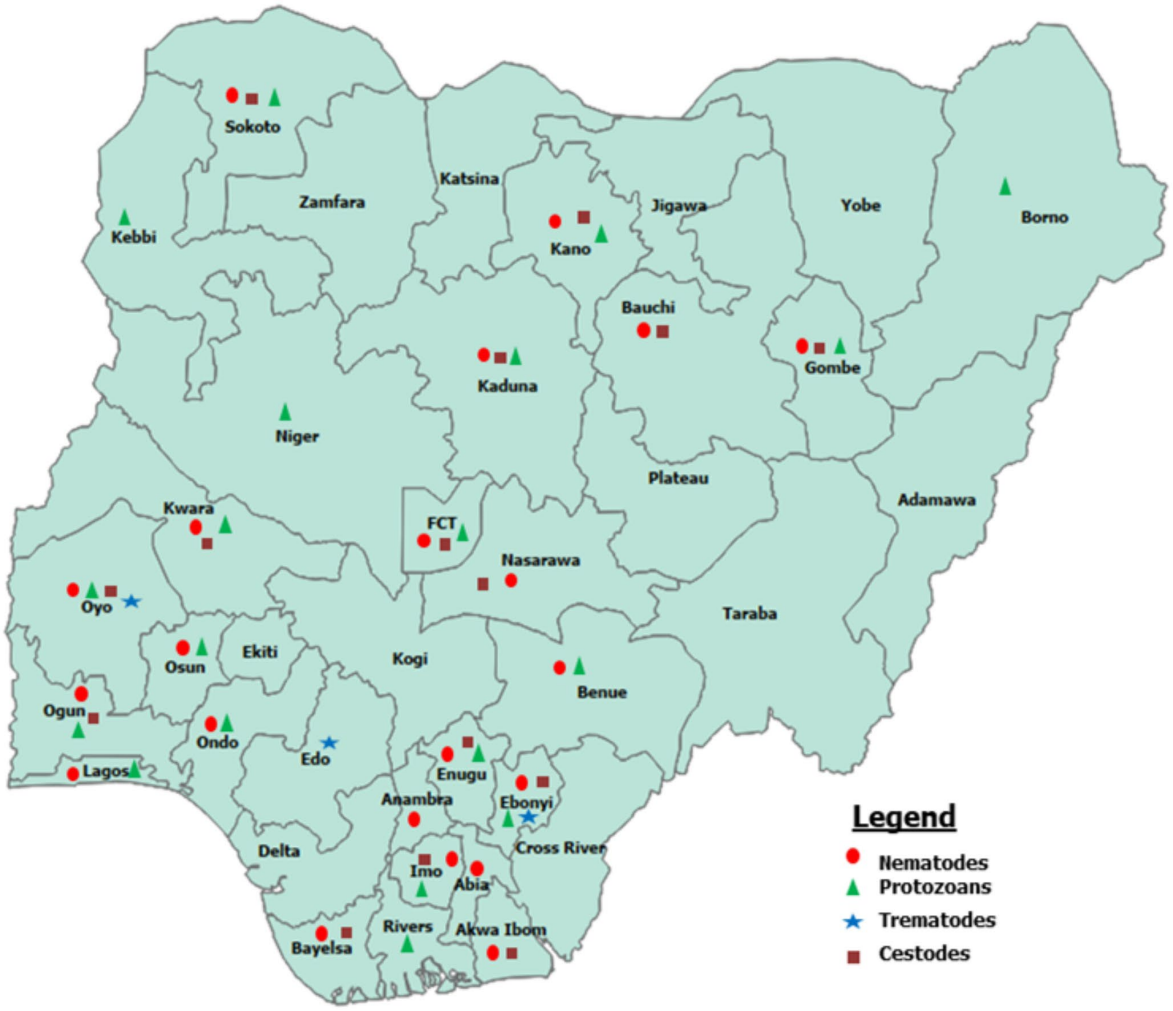
Geographical distribution of Endoparasites of birds across Nigeria

Helminths are the major cause of the disease condition helminthosis, and this condition is a major poultry issue in Nigeria and other parts of the world (Uhuo et al. 2013; Mukaratirwa and Khumaloa 2010; Baboolal et al. 2012). Worldwide, three helminth parasite classes have been reported to infect poultry birds: nematodes (Roundworms), cestodes (Tapeworms), and trematodes (Flukes) with nematodes being the most common helminth parasite of poultry, both in terms of number of species and damage they cause (Naphade 2013). These parasites are found in the intestine or faeces, especially when fresh (Fakae and Paul-Abiade 2003). Helminth parasites have been implicated as a major source of ill health, affecting the host’s metabolism, reducing feed utilization and thus growth and production among avian species in Africa and other regions of Nigeria (Uhuo et al. 2013). Gastrointestinal helminth infections cause hidden economic losses in poultry meat, eggs, and by-product production, as infected birds, especially young birds, may suffer from stunted growth, impaired development, weakened immune systems, and become susceptible to subsequent illnesses (Dauda et al. 2016). Anemia, weakness, paralysis, catarrh, diarrhea, intestinal obstruction and poor feathering are also linked to helminthoses in birds (Jegede et al. 2015; Ngongeh et al. 2014; Uhuo et al. 2013). Numerous factors, including climatic (temperature and humidity), environmental, and nutritional factors, might influence the occurrence and intensity of helminth infections (Jegede et al. 2015), high temperatures and to some extent humidity may also favour the propagation of some insects that may act as vectors for helminthes (Naphade 2013; Jegede et al. 2015). In commercial intensive poultry production, *Eimeria* spp. infections are considered as a problem compared to the free-range chickens (Njue et al. 2001). In gallinaceous birds, helminths, *Eimeria* spp. and *Cryptosporidium* spp. infections are some of the documented endo-parasites in Nigeria. Figure 2 is the map of Nigeria showing the geographical distribution of endo-parasites of poultry.

#### Cestodes

Cestodes (tapeworms) are a group of parasites that predominantly inhabit the intestinal tract of their host including poultry, with more than 1,400 different types of tapeworms been identified in domesticated poultry and wild birds (Biu and Haddabi 2005). Tapeworms belong to the phylum Platyhelminthes, class Cestoda and are all endoparasitic, hermaphroditic worms with a flat, long segmented body without an alimentary tract or body cavity (Permin and Hansen 1998; Belete et al. 2016). They are more common during the warm and/or wet seasons, when intermediate hosts are in greater abundance, and most cestodes species use intermediate hosts like ants, beetles, houseflies and oribatid mite’s to complete their life cycle in poultry buildings (Baba and Oveka 2004). In Nigeria, the most commonly diagnosed cestodes includes *Raillietina tetragona*, located in the distal jejunum, *Raillietina echinobothridia*, also located in the jejunum resulting in nodular granulomas and catarrhal enteritis (Simon and Emeritus 2005).

*Raillietina* are tapeworms, which are helminth parasites of birds. Over 37 species have been recorded across the world but only 3 namely; *R. echinobothrida*,* R. tetragona*, and *R. cesticillus* are the most important species in terms of prevalence and pathogenicity among wild and domestic birds (Cheng 1986; McDougald 2011). In Nigeria, 3 species of *Raillietina* (*R. tetragona*,* R. cesticillus* and *R. echinobothrida*) have been widely reported in poultry and their distribution and prevalence is summarized in Tables 9, 10 and 11 respectively. *Raillientina* species have been widely reported by most researchers affecting chickens (Elele et al. 2021; Nnadi and George 2010; Offiong et al. 2012; Mikial and Adamu 2008; Ngongeh et al. 2014; Jegede et al. 2018; Opara et al. 2014; Agbolade et al. 2014), turkey (Opara et al. 2014; Jegede et al. 2019), African morning dove (Omonona 2017), and wild birds (Assam et al. 2020) in Nigeria. However, *Raillientina tetragona* were majorly reported from two regions, the northwest of the country infecting chickens (Hassan et al. 2020; Luka and Ndams 2007; Imam et al. 2017), pigeons (Natala et al. 2009; Adang et al. 2008; Adang et al. 2009), wood and vinaceous doves (Adang et al. 2009a), and the northeast region in local chickens with a prevalence of 38.5% (Yoriyo et al. 2008), Muscovy ducks with a prevalence of 8.33% (Paul et al. 2015), and guinea fowl with a prevalence of 72.8% (Jajere et al. 2018). Other regions where it has been documented include the northcentral with a prevalence of 1.6% in intensively reared poultry (Ola-Fadunsin et al. 2019a), and the southeast with a prevalence of 92.5% in domestic chickens (Fakae and Paul 2003). Furthermore, *Raillientina cesticilus* and *Raillientina echinobothridia* were predominantly reported from the northwestern region (Adang et al. 2008; Luka and Ndams 2007; Udoh et al. 2014; Natala et al. 2009; Adang et al. 2009a, b; Imam et al. 2017; Yahaya et al. 2002). Other cestode species reported include *Choanotaenia infundibulum* from the northwestern region in chickens (Mikial and Adamu 2008; Luka and Ndams 2007), and turkey (Udoh et al. 2014), as well as in Northeastern region in local chickens (Yoriyo et al. 2008), and guinea fowl (Jajere et al. 2018).

**Table 9:** Distribution and prevalence of *Raillientina tetragona* in poultry across the geopolitical zones in Nigeria

Geographical region	Location	Avian type	Number examined	No. of positive samples	Prevalence (%)	References
North-West	Kaduna	Wood dove	40	1	2.50	Adang et al. (2009a)
		Vinaceous dove	10	0	0.00	
North-West	Kaduna	Pigeon	30	1	3.30	Adang et al. (2009)
North-West	Kano	Local chicken	50	11	30.60	Imam et al. (2017)
South-East	Enugu	Domestic chicken	80	74	92.50	Fakae and Paul (2003)
North-West	Kaduna	Domestic pigeon	240	65	27.10	Adang et al. (2008)
North-East	Bauchi	Local chicken	200	77	38.50	Yoriyo et al. (2008)
North-East	Gombe	Muscovy ducks	600	50	8.33	Paul et al. (2015)
North-East	Gombe	Guinea fowl	600	437	72.80	Jajere et al. (2018)
North-Central	Kwara	Poultry (intensive)	502	8	1.60	Ola-Fadunsin et al. (2019a)
North-West	Kaduna	Domestic chicken	92	22	23.90	Luka and Ndams (2007)
North-West	Zaria	Domestic pigeon	250	8	4.90	Natala et al. (2009)
North-West	Kaduna	Poultry	245	1	0.40	Hassan et al. (2020)

**Table 10:** Distribution and prevalence of *Raillientina cesticillus* in poultry across the geopolitical zones in Nigeria

Geographical region	Location	Avian type	Number examined	No. of positive samples	Prevalence (%)	References
North-West	Zaria, Kaduna	Domestic pigeon	240	1	0.45	Adang et al. (2008)
North-East	Bauchi	Local chicken	200	21	10.50	Yoriyo et al. (2008)
South-South	Akwa Ibom	Chicken	150	25	16.70	Ozougwu et al. (2021)
North-East	Gombe	Guinea fowl	600	300	50.00	Jajere et al. (2018)
North-West	Kaduna	Domestic chicken	92	9	9.80	Luka and Ndams (2007)
North-West	Kaduna	Turkey	196	5	2.60	Udoh et al. (2014)
North-West	Kaduna	Domestic pigeon	250	5	3.00	Natala et al. (2009)
North-West	Kaduna	Wood dove	40	4	10.00	Adang et al. (2009a)
		Vinaceous dove	10	2	20.00	
North-West	Kaduna	Pigeon	30	8	26.70	Adang et al. (2009)
North-West	Kano	Local chicken	50	6	16.70	Imam et al. (2017)
North-West	Kano	Guinea fowl	100	8	8.00	Yahaya et al. (2002)

**Table 11:** Distribution and prevalence of *Raillientina Echinobothridia* in poultry across the geopolitical zones in Nigeria

Geographical region	Location	Avian type	Number examined	No. of positive samples	Prevalence (%)	References
North-West	Zaria	Domestic chicken	92	12	13.00	Luka and Ndams (2007)
North-West	Zaria	Domestic chicken	250	19	7.60	Natala et al. (2009)
North-West	Zaria	Wood dove	40	5	12.50	Adang et al. (2009a)
		Vinaceous dove	10	0	0.00	
North-West	Kano	Local chicken	50	9	25.00	Imam et al. (2017)
North-West	Sokoto	Guinea fowl	200	64	34.40	Attah et al. (2013)
		Chicken	200	90	56.90	
North-West	Kano	Guinea fowl	100	18	18.00	Yahaya et al. (2002)
North-West	Zaria, Kaduna	Domestic pigeon	240	26	10.60	Adang et al. (2008)
North-East	Bauchi	Local chicken	200	84	42.00	Yoriyo et al. (2008)
South-East	Ebonyi	Local chicken	150	31	20.10	Uhuo et al. (2013)
North-East	Gombe	Muscovy ducks	600	62	10.33	Paul et al. (2015)
North-East	Gombe	Guinea fowl	600	404	67.30	Jajere et al. (2018)

#### Nematodes

When it comes to species diversity and the amount of damage caused, nematodes stand out as the most common and problematic helminth infection of birds. The nematodes that afflict poultry are parasitic, unsegmented worms that belong to the phylum Nemathelminthes, class Nematoda (Belete et al. 2016). Nematode is the most common internal parasites of birds and includes *Ascaridia galli* (intestine), *Heterakis gallinarum* (ceca) and various *Capillaria* species (crop→intestine) found through the digestive (Simon and Emeritus 2005; Belete et al. 2016). *Ascaridia galli* is the most common parasite of poultry, followed by the caecal nematode *Heterakis gallinarum*, and they both often occurs as a mixed infection (Höglund et al. 2023), and they are widely distributed across all the geopolitical zones of Nigeria.

*Ascaridia galli* is a parasitic roundworm and the most prevalent and pathogenic species, especially in domestic fowl. It is the largest nematode of birds and causes a disease condition of poultry referred to as ascaridiasis, which is associated with heavy worm infection, particularly in chicken and turkeys (Belete et al. 2016). In Nigeria, *Ascaridia galli* is the most widely reported gastrointestinal nematode of poultry and majority of the reports have been in, chickens from the north central regions (Ogbaje et al. 2012; Ola-Fadunsin et al. 2019b; Jegede et al. 2015, 2019; Mohammed et al. 2019; Ombugadu et al. 2021), Muscovy ducks and guinea fowl from the northeast region (Paul et al. 2015; Jajere et al. 2018), in chickens, guinea fowl, pigeon, and turkey from the northwestern region (Imam et al. 2017; Attah et al. 2013; Yahaya et al. 2022; Adang et al. 2008; Luka and Ndams 2007; Udoh et al. 2014; Natala et al. 2009; Mikial and Adamu 2008; Hassan et al. 2020). In the Southeast region, *A. galli* has been reported in chickens, commercial quails, turkey and street pigeon (Nnadi and George 2010; Fakae and Paul 2003; Ozougwo et al. 2021; Uhuo et al. 2013; Opara et al. 2014 and 2012; Onyeabor and Uwalaka 2021; Ngongeh et al. 2014), and in the south south region, this nematode has been documented among chickens (Elele et al. 2021; Offiong et al. 2013; Johnson et al. 2019), while in the southwest, it’s been reported among domestic chickens, ducks and mourning doves (Ekpo et al. 2010; Afolabi et al. 2016; Omonona et al. 2017; Adejinmi and Oke 2011; Agbolade et al. 2014). An overview of some of the documented reports is summarized in Table 12.

**Table 12:** Distribution and prevalence of *Ascaridia galli* in poultry across the geopolitical zones in Nigeria

Geographical region	Location	Avian type	Reported predilection site	Number Examined	No. of positive samples	Prevalence (%)	References
South-South	Yenagoa, Bayelsa	Free range chicken	-	35	28	4.40	Elele et al. (2021)
South-East	Enugu	Chicken	-	261	45	17.20	Nnadi and George (2010)
South-Eest	Abeokuta, Ogun	Free range chicken	Small intestine	20	12	60.00	Ekpo et al. (2010)
North-West	Kano	Local chicken	Small intestine	50	5	13.90	Imam et al. (2017)
South-East	Nsukka, Enugu	Domestic chicken	-	80	1	1.25	Fakae and Pau (2003)
South-South	Akwa Ibom	Local chicken	-	200	92	46.00	Offiong et al. (2013)
North-West	Sokoto	Guinea fowl	-	200	0	0.00	Attah et al. (2013)
		Chicken	-	200	34	21.50	
North-West	Garin dua, Kano	Guinea fowl	-	100	25	25.00	Yahaya et al. (2022)
North-West	Zaria, Kaduna	Domestic pigeon	Duodenum/Ileum	240	8	3.30	Adang et al. (2008)
South-East	Akwa, Anambra	Chicken	-	150	20	13.30	Ozougwo et al. (2021)
South-East	Abakaliki, Ebonyi	Local chicken	-	150	73	48.70	Uhuo et al. (2013)
North-Central	Makurdi, Benue	Local chicken, broilers, layers	-	440	165	37.50	Ogbaje et al. (2012)
North-East	Gombe	Muscovy ducks	Small intestine	600	514	85.67	Paul et al. (2015)
South-South	Abak, Akwa Ibom	Chicken	-	270	113	42.00	Johnson et al. (2019)
North-East	Gombe	Guinea fowl	Caecum	600	340	56.70	Jajere et al. (2018)
North-Central	Kwara	Poultry (Intensive)	-	502	18	3.60	Ola-Fadunsin et al. (2019a)
South-West	Akure, Ondo	Domestic chicken	-	327	23	7.00	Afolabi et al. (2016)
North-West	Samaru, Zaria	Domestic chicken	Large and small intestine	92	40	43.80	Luka and Ndam (2007)
North-Central	Gwagwalada, Abuja	Local and Exotic chicken	-	280	43	15.40	Jegede et al. (2015)
North-West	Kaduna	Turkey	Small intestine	196	51	26.00	Udoh et al. (2014)
South-East	Owerri, Imo	Chicken and turkey	-	5600	1568	28.00	Opara et al. (2014)
North-West	Zaria, Kaduna	Domesticated pigeon	-	250	3	1.20	Natala et al. (2009)
South-West	Ibadan, Oyo	Mourning dove	-	30	8	26.67	Omonona et al. (2017)
South-East	Umuahia, Abia	Commercial quails	-	300	63	37.70	Onyeabor and Uwalaka (2021)
North-West	Sokoto	Chicken	-	150	28	18.66	Mikial and Adamu (2008)
South-East	Nsukka, Enugu	Chicken	-	305	68	22.30	Ngongeh et al. (2014)
North-West	Kaduna	Poultry	-	245	5	2.00	Hassan et al. (2020)
South-West	Ibadan	Domestic ducks	-	175	82	46.80	Adejinmi and Oke (2011)
South-East	Owerri, Imo	Street pigeon	-	150	21	14.00	Opara et al. (2012)
South-West	Ijebu north, Ogun	Domestic fowl	-	133	14	10.50	Agbolade et al. (2014)
North-Central	Gwagwalada, Abuja	Domestic chicken	-	108	38	35.20	Mohammed et al. (2019)
North-Central	Gwagwalada, Abuja	Local and Exotic turkey	-	211	47	22.27	Jegede et al. (2019)
North-Central	Lafia, Nasarawa	Poultry	-	100	7	7.00	Ombugadu et al. (2021)
North-Central	Ilorin, Kwara	Chickens	-	597	180	30.15	Ola-Fadunsin et al. (2019b)

*Heterakis gallinarum* is a small, white caecal worm that thrives in the ceca of various species of gallinaceous birds. This helmith is recognized as an economically important parasite by the poultry industry because its ovum serves as the vector for the protozoal parasite *Histomonas meleagridis*, the cause of histomonosis in poultry (Cupo and Beckstead 2019). *Heterakis gallinarum* is a heavily prevalent poultry parasite that is well distributed in Nigeria across all the geopolitical zones and have been registered in the northwest region (Imam et al. 2017; Attah et al. 2013; Yahaya et al. 2022; Adang et al. 2008; Mikail and Adamu 2008; Luka and Ndam 2007; Udoh et al. 2014), north central (Ogbage et al. 2012Ola-Fadunsin et al. 2019a; Jegede et al. 2015, 2019; Mohammed et al. 2019), south east region (Nnadi and George 2010; Onyeabor and Uwalaka 2021; Ozougwu et al. 2021; Uhuo et al. 2013; Ngongeh et al. 2014), and South-West region of Nigeria (Ekpo et al. 2010; Esan et al. 2018; Afolabi et al. 2016; Adejinmi and Oke 2011; Agbolade et al. 2014). A summary of all the documented reports is presented in Table 13.

**Table 13:** Distribution and prevalence of *Heterakis gallinarum* in poultry across the geopolitical zones in Nigeria

Geographical region	Location	Avian type	Reported predilection site	Number Examined	No. of positive samples	Prevalence (%)	References
South-South	Yenagoa, Bayelsa	Domestic chicken	-	35	21	60.00	Elele et al. (2021)
South-East	Enugu	Chicken	-	261	33	12.64	Nnadi and George (2010)
South-West	Abeokuta, Ogun	Free rang chicken	Caecum	20	12	60.00	Ekpo et al. (2010)
North-West	Kano	Local chicken	Caecum	50	5	10.00	Imam et al. (2017)
South-South	Akwa Ibom	Local chicken	-	200	62	31.00	Offiong et al. (2013)
North-West	Sokoto	Guinea fowl	-	200	30	16.10	Attah et al. (2013)
		Chicken	-	200	22	13.90	
North-West	Garin dua, Kano	Guinea fowl	-	100	15	15.00	Yahaya et al. (2022)
South-East	Umuahia, Abia	Commercial Quails	-	300	41	24.60	Onyeabor and Uwalaka (2021)
South-West	Ibadan, Oyo	Water fowl, duck and geese	-	100	43	43	Esan et al. (2018)
North-West	Zaria, Kaduna	Domestic pigeon	Caeca	240	8	3.30	Adang et al. (2008)
South-East	Akwa, Anambra	Chicken	-	150	18	12.00	Ozougwu et al. (2021)
South-East	Abakaliki, Ebonyi	Local chicken	-	150	42	28.00	Uhuo et al. (2013)
North-Central	Makurdi, Benue	Local chicken, broilers and layers	-	440	122	27.70	Ogbage et al. (2012)
North-East	Gombe	Muscovy ducks	Caecum	600	477	79.50	Paul et al. (2015)
South-South	Abak, Akwa Ibom	Chicken	-	270	83	31.00	Johnson et al. (2019)
North-West	Sokoto	Chicken	-	150	43	28.66	Mikial and Adamu (2008)
North-East	Gombe	Guinea fowl	Caecum	600	103	17.20	Jajere et al. (2018)
North-Central	Kwara	Poultry (intensive)	-	502	33	6.60	Ola-Fadunsin et al. (2019a)
South-East	Nsukka, Enugu	Chicken	-	305	1	0.60	Ngongeh et al. (2014)
South-West	Akure	Domestic chicken	-	327	6	1.80	Afolabi et al. (2016)
North-West	Samaru, Kaduna	Domestic chicken	Caecum and small intestine	92	31	33.70	Lucas and Ndam (2007)
North-Central	Gwagwalada	Local and exotic chicken	-	280	15	5.40	Jegede et al. (2015)
North-West	Kaduna	Turkey	Caecum	196	2	1.00	Udoh et al. (2014)
South-West	Ibadan, Oyo	Domestic ducks	-	175	41	23.40	Adejinmi and Oke (2011)
South-West	Ijebu north, Ogun	Domestic fowl	-	133	9	6.80	Agbolade et al. (2014)
North-Central	Gwagwalada, Abuja	Domestic chicken	-	108	6	5.60	Mohammed et al. (2019)
North-Central	Gwagwalada, Abuja	Local and exotic turkey	-	100	4	25.00	Jegede et al. (2019)
North-Central	Ilorin, Kwara	Chicken, guinea fowl, duck, turkey, pigeon	-	597	148	24.79	Ola-Fadunsin et al. (2019b)

*Capillaria* species are nematodes parasites that are found throughout the intestinal tract of poultry including the crop, oesophagus and caeca of birds and about six species have been recognized to parasitize domesticated and wild wilds (Belete et al. 2016). *Capillaria* species was predominately reported from all geopolitical regions with the exception the northeast. It was reported in the southwestern region among chickens, ducks, and mourning doves (Afolabi et al. 2016; Otegbade and Morenikeji 2014; Omonona et al. 2017; Adejinmi and Oke 2011; Agbolade et al. 2014), the north central region among local and exotic chickens and turkey (Jegede et al. 2015, 2019), the south east region among chickens and commercial quails (Nnadi and George 2010; Onyeabor and Uwalaka 2021) and in the South south region among local chickens (Elele et al. 2021; Offiong et al. 2013). A summary of the some of the epidemiological studies reporting the prevalence of *Capillaria* species of poultry is presented in Table 14.

**Table 14:** Distribution and prevalence of *Capillaria species* in poultry across the geopolitical zones in Nigeria

Geographical region	Location	Avian type	Reported predilection site	Number Examined	No. of positive samples	Prevalence (%)	References
South-South	Yenagoa, Bayelsa	Domestic chicken	-	35	14	22.20	Elele et al. (2021)
South-East	Enugu	Chicken	-	261	15	5.70	Nnadi and George (2010)
South-South	Akwa Ibom	Local chicken	-	200	58	29	Offiong et al. (2013)
South-East	Umuahia, Abia	Commercial quails	-	300	33	19.8	Onyeabor and Uwalaka (2021)
North-West	Sokoto	Chicken	-	150	6	4	Mikial and Adamu (2008)
South-West	Akure, Ondo	Domestic chicken	-	327	3	0.9	Afolabi et al. (2016)
North-Central	Gwagwalada, Abuja	Local and exotic chicken	-	280	5	1.8	Jegede et al. (2015)
North-West	Kaduna	Turkey	Oesophagus	196	1	0.5	Udoh et al. (2014)
North-West	Kaduna	Wild birds	-	357	2	0.6	Assam et al. (2020)
South-West	Ibadan, Osun, Ilorin, lagos, Abeokuta	Birds spp	-	178	25	14.1	Otegbade and Morenikeji (2014)
South-West	Ibadan, Oyo	Mourning dove	-	30	10	33.33	Omonona et al. (2017)
South-West	Ibadan, Oyo	Domestic ducks	-	175	38	21.7	Adejinmi and Oke (2011)
South-West	Ijebu north, Ogun	Domestic fowl	-	133	12	9	Agbolade et al. (2014)
North-Central	Gwagwalada, Abuja	Local and exotic turkey	-	100	26	26	Jegede et al. (2019)

*Subulura brumpti* is a common gastrointestinal nematode of chickens, turkeys, guineafowls, ducks, pheasants, grouse and quails (Soulsby 1982), with the adult worms occurring in the lumen of the caeca and are quite similar in shape and size to *Heterakis* spp. (Urquart et al. 1996). *Subulura brumpti* was predominately reported from the northern region (Yoriyo et al. 2008; Udoh et al. 2014; Paul et al. 2015; Ola-Fadunsin et al. 2019a; Jegede et al. 2019; Hassan et al. 2020), and details of other reports across the country is summarized in Table 15.

**Table 15:** Distribution and prevalence of *Subulura brumpti* in poultry across the geopolitical zones in Nigeria

Geographical region	Location	Avian type	Reported predilection site	Number Examined	No. of positive samples	Prevalence (%)	References
South-East	Enugu	Chicken	-	261	6	2.3	Nnadi and George (2010)
South-East	Nsukka, Enugu	Domestic chicken	Caecum	80	13	16.3	Fakae and Paul-Abiade (2003)
North-West	Sokoto	Guinea fowl	-	200	11	5.9	Attah et al. (2013)
		Chicken	-	200	0	0	
North-East	Bauchi	Local chicken	Caecum	200	31	15.5	Yoriyo et al. (2008)
North-East	Gombe	Muscovy ducks	Caecum	600	242	40.33	Paul et al. (2015)
North-Central	Kwara	Poultry (intensive)	-	502	2	0.4	Ola-Fadunsin et al. (2019a)
North-West	Kaduna	Turkey	Large intestine	196	7	3.6	Udoh et al. (2014)
North-West	Kaduna	Poultry	-	245	1	0.4	Hassan et al. (2020)
North-Central	Gwagwalada, Abuja	Local and exotic turkey	-	100	6	6	Jegede et al. (2019)

#### Trematodes

Infections with trematodes are not particularly prevalent in domestic chickens (Hodasi 1978). However, Uhuo et al. (2013) had reported a prevalence of 10.7% (*Prosthogonimus species*) in **Ebonyi state (southeast).**

#### Coccidia protozoan parasites

Coccidia infection is common in local and commercial poultry and causes losses through lowered productivity and deaths. *Cryptosporidium* species is a coccidian protozoan parasite that have been documented to inhabit the gastrointestinal tract of avians. In Nigeria, *Cryptosporidium* species has been documented in domestic duck (Adejinmi and Oke 2011), free range birds (Olonisakin and Olusi 2021; Danladi and Garba 2021), exotic birds (Jegede et al. 2019) with prevalence’s ranging from 6.4 to 34.0%. It has been reported in poultry mostly from the southwestern region (Adejinmi and Oke 2011; Adeyemi et al. 2019; Olonisakin and Olusi 2021) (Table 16).

**Table 16:** Distribution and prevalence of *Cryptosporidium* species in poultry across the geopolitical zones in Nigeria

Geographical region	Location	Avian type	Number Examined	No. of positive samples	Prevalence (%)	References
South-West	Ibadan, Oyo	Domestic ducks	175	27	15.40	Adejinmi and Oke (2011)
North-Central	Gwagwalada, Abuja	Local and exotic turkey	100	34	34.00	Jegede et al. (2019)
South-West	Lagos	Local and exotic chicken	100	18	18.00	Adeyemi et al. (2019)
South-West	Akure, Ondo	Poultry (intensive)	247	27	10.90	Olonisakin and Olusi (2021)
		Free range birds	109	25	13.20	
North-West	Kano	Avian (turkey, chicken, duck, pigeon, guinea fowl	330	21	6.40	Mustapha et al. (2016)
North-West	Kebbi	Free nature chicken	300	75	25.00	Danladi and Garba (2021)

Avian coccidiosis is a major parasitic disease of poultry (Adewole 2012; Garbi et al. 2015). The disease has been shown to be common and significant in intensively managed commercial poultry farms in Nigeria especially where management or hygienic standards are compromised (Mohammed and Sunday 2015). Cases of coccidiosis were reported across all the geopolitical zones, mostly from the southwest (Afolabi et al. 2016; Omonona et al. 2017; Adejinmi and Oke 2011; Agbolade et al. 2014; Idowu et al. 2019; Adejinmi et al. 2019; Ola-Fadunsin 2017; Majaro 1980; Otegbade and Morenikeji 2014), north-central (Jegede et al. 2019; Ola-Fadunsin et al. 2019b; Comfort et al. 2014; Babatude et al. 2016; Otegbade and Morenikeji 2014), and north-east regions (Grema et al. 2014; Jemimah et al. 2017; Adang and Isa 2016; Lawal et al. 2016a, b). Details of the distribution of avian coccidiosis across all the geopolitical zones of Nigeria are presented in Table 17. Generally, seven species of *Eimeria* are widely recognized as the causative agents of coccidiosis in chickens, of which *Eimeria tenella*,* Eimeria necatrix*,* Eimeria maxima* and *Eimeria brunetti* are the highly pathogenic, while *E. acervulina* and *E. mitis* are less pathogenic, whilst *E. praecox* is regarded least pathogenic (McDougald 2003; Shirley et al. 2005; Conway and McKenzie 2007; Taylor et al. 2007). Transmission of *Eimeria* is mediated via an environmentally resistant oocyst and infection occurs when a susceptible chicken ingests the sporulated oocysts. Infective oocysts could be spread by contaminated equipment, dust, people, rodents, wild birds as well as insects (Champman 1997; Majaro 2001).

**Table 17:** Distribution and prevalence of avian coccidiosis in poultry across the geopolitical zones in Nigeria

Geographical region	Location	Avian type	Number Examined	No. of positive samples	Prevalence (%)	References
South-West	Akure, Ondo	Domestic chicken	327	25	7.70	Afolabi et al. (2016)
North-West	Kaduna	Turkey	196	44	22.40	Udoh et al. (2014)
South-West	Ibadan, Oyo	Mourning dove	30	1	3.33	Omonona et al. (2017)
South-West	Ibadan, Oyo	Domestic ducks	175	60	34.30	Adejinmi and Oke (2011)
South-East	Owerri, Imo	Street pigeon	150	43	28.60	Opara et al. (2012)
South-West	Ijebu north, Ogun	Domestic fowl	133	9	6.80	Agbolade et al. (2014)
North-Central	Gwagwalada, Abuja	Local and exotic turkey	100	41	41.00	Jegede et al. (2019)
North-Central	Ilorin, Kwara	Avian species	597	196	32.83	Ola-Fadunsin et al. (2019b)
South-West	Lagos	Chicken	60	19	31.70	Idowu et al. (2019)
		Guinea fowl	60	21	35.00	
South-West	Lagos	Local and exotic chicken	100	22	22.00	Adejinmi et al. 2019
South-East	Nsukka, Enugu	Domestic chicken	900	158	39.50	Okwuonu et al. (2021)
North-East	Gombe	Chicken	5102	581	11.40	Grema et al. (2014)
South-South	Eligbo, Rivers	Domestic birds	200	84	42.00	Nzeako et al. (2017)
South-West	Osun	(Chicken, turkey, ducks, ostrich	5544	2292	41.30	Ola-Fadunsin (2017)
North-West	Kano	Pigeon	144	28	19.44	Mohammed et al. (2017)
North-East	Gombe	Desi and broiler chicken	100	21	21.00	Jemimah et al. (2017)
North-East	Maiduguri, Borno	Domestic and exotic chicken	600	191	31.80	Lawal et al. (2016a)
North-Central	Minna, Niger	Chicken	200	56	28.00	Eke et al. (2016)
North-East	Jere, Borno	Domesticated chicken (local and exotic)	430	54	12.60	Lawal et al. (2016b)
South-West	Oyo	Poultry	500	430	86.00	Majaro (1980)
North-East	Gombe	Local chicken	150	64	42.70	Adang and Isa (2016
North-Central	Gwagwalada, Abuja	Poultry bird	200	138	69.00	Comfort et al. (2014)
North-Central	Gwagwalada, Abuja	Exotic chicken	50	9	18.00	Babatunde et al. (2016)
		Local chicken	50	16	32.00	
South-West, North-Central	Ibadan, Osun, Ilorin, Abeokuta, Lagos	Birds	178	14	7.90	Otegbade and Morenikeji (2014)

The most frequently reported species of *Eimeria* reported include, *E. acervulina*,* E. tenella*, and *E. maxima* from the north east region among chickens (Lawal et al. 2016a, b; Adang and Isa 2016), north-central (Comfort et al. 2014), and north west region among domestic chickens (Luka and Ndams 2007), south west region among broiler chickens (Majaro 1981), and southeast region infecting exotic chickens (Ngele 2017). Other *Eimeria* species reported includes, *E. necatrix* (Luka and Ndams 2007; Ngele 2017; Majaro 1981; Lawal et al. 2016a, b; Adang and Isa 2016, *E. mitis* (Luka and Ndams 2007, with a prevalence of 9.8% from the north west region while in the south east region, the documented prevalence was 3% (Ngele 2017), and a prevalence of 4.75% was registered in south west Nigeria by Majaro (1981). *Eimeria brunetti* with a prevalence of 10.9% was registered in north west region (Luka and Ndams 2007), while a prevalence of 8.8% was documented in south west region (Majaro et al. 1981). Specific details regarding the prevalence and distribution of *Eimeria* species across the geopolitical zones in Nigeria is presented in Table 18.

**Table 18:** Distribution and prevalence of *Eimeria* species in poultry across the geopolitical zones in Nigeria

Geographical region	Location	Avian species	Number Examined	Prevalence (%)							
				Eimeria acervulina	Eimeria mitis	Eimeria tenella	Eimeria brunetti	Eimeria maxima	Eimeria mivatis	Eimeria necatrix	References
North-West	Samaru, Kaduna	Domestic chicken	92	8 (8.7%)	9 (9.8%)	26 (28.3%)	10 (10.9%)	3 (3.3%)	2 (2.2%)	4 (4.4%)	Luka and Ndams (2007)
South-East	Afikpo, Ebonyi	Exotic chicken	372	25 (6.7%)	11 (3%)	47 (12.6%)	-	12 (3.2%)	-	30 (8.1%)	Ngele (2017)
South-West	Ibadan, Oyo	Broiler chicken	240	36.66%	4.75%	25.82%	8.80%	6.25%	3.25%	14.47%	Majaro (1981)
North-East	Jere, Borno	Domestic chicken	54	15 (27.8%)	-	13 (24.1%)	-	3 (5.6%)	-	23 (42.6%)	Lawal et al. (2016a, b)
North-East	Gombe	Local chicken	150	18 (28.1%)	-	25 (39.1%)	-	9 (14.1%)	-	12 (18.8%)	Adang and Isa (2016)
North-Central	Gwagwalada, Abuja	Poultry birds	200	26 (13%)	-	28 (14%)	-	84 (42%)	-	-	Comfort et al. (2014)

### Haemoparasites

Avian malaria parasites (specifically *Plasmodium* spp.) and other haemosporidians (such as *Haemoproteus* and *Leucocytozoon* spp.) belong to the order Apicomplexa and are a diverse group of protozoan parasites recovered from all avian clades and are vector-transmitted blood parasites (Valkiūnas 2005). Haemosporidian parasites of the genera *Plasmodium*,* Leucocytozoon*, and *Haemoproteus* are one of the most prevalent and widely studied groups of protozoan parasites infecting birds (Permin et al. 1998; Clark et al. 2009). Avian haemosporidian parasites are heteroxenous, with transmission possible with different groups of dipteran vectors (Valkiūnas 2005), utilizing Mosquitoes, Midges, *Simulium*,* Culicodes*, or Hippoboscid as their arthropod vectors (Soulsby 1982). Avian haemoparasites have been reported in several countries including Nigeria (Poulsen et al. 2000; Permin et al. 2002; Njunga 2003; Sadiq et al. 2003; George et al. 2004; Schultz and Whittington 2005; Nnadi and George 2010; Karamba et al. 2012; Opara et al. 2012; Usman et al. 2012; Gimba et al. 2014).

*Plasmodium* species have been reported among avian including local and exotic chickens, mourning doves and village weavers in Nigeria (Sadiq et al. 2003; Omonona et al. 2017; Olayemi et al. 2014; Agbolade et al. 2014; Idowu et al. 2019; Adeyemi et al. 2019; Lawal et al. 2016a, b; 2020; 2021; 2022). An overview of some of the reports that have been documented across Nigeria is summarized in Table 19. *Haemoproteus* species another haemoparasite has been reported by researchers predominately from the northern region among indigenous chickens and pigeons (Natala et al. 2009; Mohammed et al. 2019; Lawal et al. 2016a, b; 2020; 2021; 2022) and their distribution and prevalence data are presented in Table 20. Lastly, *Leucocytozoon* species, another haemosporidian of poultry was reported both in the north and south parts of Nigeria (Sadiq et al. 2003; Omonona et al. 2017; Olayemi et al. 2014; Idowu et al. 2019; Lawal et al. 2020, 2021, 2022), and the summary of the reports are presented in Table 21.

**Table 19:** Distribution and prevalence of *Plasmodium* species in poultry across the geopolitical zones in Nigeria

Geographical region	Location	Avian type	Number Examined	No. of positive samples	Prevalence (%)	References
North-East	Gombe	Chicken	1600	163	10.20	Lawal et al. (2022)
			220	63	28.60	
South-East	Nsukka, Enugu	Chicken, duck, pigeon, turkey	109	18	16.50	Amaka et al. (2018)
North-East	Yalmaltu deba, Gombe	Free range chicken	400	55	13.80	Lawal et al. (2020)
North-East	Kwami, Gombe	Domestic chicken	346	41	11.80	Lawal et al. (2021)
North-West	Kano	Domesticated birds	102	12	12.24	Karamba et al. (2012)
		Wild birds	116	8	9.28	
North-Central	Makurdi, Benue	Chicken	220	48	21.60	Ogbaje et al. (2019)
South-West	Ibadan, Oyo	Domestic chicken	150	48	32.00	Sadiq et al. (2003)
South-West	Ibadan, Oyo	Mourning dove	30	10	40.00	Omonona et al. (2017)
South-East	Owerri, Imo	Street pigeon	150	30	25.00	Opara et al. (2012)
North-Central	Gwagwalada, Abuja	Domestic chicken	108	59	54.60	Mohammed et al. (2019)
South-West	Ibadan, Oyo	Domestic weaver	30	5	16.67	Olayemi et al. (2014)
South-West	Ijebu north, Ogun	Domestic fowl	133	1	0.80	Agbolade et al. (2014)
South-West	Lagos	Chicken	60	14	23.30	Idowu et al. (2019)
		Guinea fowl	60	9	15.00	
North-East	Gombe	Domestic chicken	530	69	13.00	Lawal et al. (2021)
North-East	Maiduguri, Borno	Domestic chicken	200	10	29.40	Lawal et al. (2016a, b)
South-West	Lagos	Local and exotic chicken	100	16	16.00	Adeyemi et al. (2019)
North-West	Zaria, Kaduna	Domesticated pigeon	250	2	0.80	Natala et al. (2009)
North-West	Sokoto	Free range chicken	100	12	12.00	Usman et al. (2012)

**Table 20:** Distribution and prevalence of *Leucocytozoon species* in poultry across the geopolitical zones in Nigeria

Geographical region	Location	Avian type	Number Examined	No. of positive samples	Prevalence (%)	References
South-West	Ibadan, Oyo	Domestic chicken	150	30	20.00	Sadiq et al. (2003)
North-West	Zaria, Kaduna	Domestic chicken	250	16	6.40	Natala et al. (2009)
South-West	Ibadan, Oyo	Mourning dove	30	6	24.00	Omonona et al. (2017)
North-Central	Gwagwalada, Abuja	Domestic chicken	108	1	0.90	Mohammed et al. (2019)
South-West	Ibadan, Oyo	Domestic weaver	30	7	23.33	Olayemi et al. (2014)
South-West	Lagos	Chicken	60	4	6.70	Idowu et al. (2019)
North-East	Gombe	Domestic chicken	530	4	0.80	Lawal et al. (2021)
North-East	Gombe	Chicken	1600	5	0.30	Lawal et al. (2022)
North-East			220	33	15.00	
North-East	Gombe	Free range chicken	400	3	0.80	Lawal et al. (2020)
North-Central	Makurdi, Benue	Chicken	220	1	0.80	Ogbaje et al. (2019)

**Table 21:** Prevalence and distribution of *Haemoproteus species* in poultry across the geopolitical zones in Nigeria

Geographical region	Location	Avian type	Number Examined	No. of positive samples	Prevalence (%)	References
North-East	Gombe	Domestic chicken	530	27	5.10	Lawal et al. (2021)
North-East	Maiduguri, Borno	Domestic chicken	200	18	52.90	Lawal et al. (2016a, b)
North-East	Gombe	Chicken	1600	93	5.80	Lawal et al. (2022)
			220	45	20.50	
North-East	Yalmaltu deba, Gombe	Free range chicken	400	27	6.80	Lawal et al. (2020)
North-East	Kwami, Gombe	Domestic chicken	346	23	6.60	Lawal et al. (2021)
South-West	Ibadan, Oyo	Domestic chicken	150	2	1.30	Sadiq et al. (2003)
South-West	Ibadan, Oyo	Mourning dove	30	6	24.00	Omonona et al. (2017)
South-East	Owerri, Imo	Street pigeon	150	90	75.00	Opara et al. (2012)
North-Central	Gwagwalada, Abuja	Domestic chicken	108	1	0.90	Mohammed et al. (2019)
North-West	Zaria, Kaduna	Domesticated pigeon	250	39	15.20	Natala et al. (2009)
South-West	Ibadan, Oyo	Domestic weaver	30	19	63.33	Olayemi et al. (2014)
South-West	Lagos	Chicken	60	2	3.30	Idowu et al. (2019)
		Guinea fowl	60	2	3.30	

## Discussion

Avian populations are often affected by external and internal parasites, which cause negative impacts on their well-being, reproductive capacity and survival rates (Ola-Fadunsin et al. 2019a; Ghafouri et al. 2023; Ghaniei et al. 2023). Poultry are host to a wide range of parasitic organisms, including avian-specific cestodes, trematodes, nematodes, protozoa and arthropods such as fleas, lice, ticks and mites.

Several species of hematophagous ectoparasites were identified in poultry across Nigeria and they include *Argas persicus*,* Menacanthus stramineus*, *Lipeurus caponis*,* Menopon gallinae*,* Columbicola columbae*,* Knemidocoptes mutans*,* Dermanysuss gallinae* and *Echidnophaga gallinacean* at varying prevalence across several classes of poultry. Infestation with these external parasites affect poultry health by causing skin irritation, inducing stress, toxicosis, allergies, blood loss, dermatitis, damage to the feathers and decrease reproductive capacity (Atkinson et al. 2009; Mullens et al. 2009). Furthermore, hematophagous ectoparasites are responsible for the transmission of several pathogens such as *Bartonella* spp., *Anaplasma* spp., *Salmonella gallinarum*, *Borrelia* spp., and *Coxiella burneti* (Lafri et al. 2017; Pugliese et al. 2019; Hosseini-Chegeni and Kayedi 2020). The prevalence of *A. persicus* ranges from 2.67% in Borno to 62.20% in Sokoto states, all in northern Nigeria. This soft tick is known to parasitize the chickens for blood-feeding at night and their larvae can cause paralysis in birds (Rosenstein 1976). The larvae may also be responsible for the transmission of avian spirochaetosis (Abdu 1987). Furthermore, the DNA of several bacteria such as *Rickettsia hoogstraalii*, *Borrelia* spp., *Anaplasma* spp., *Bartonella* spp. and *Coxiella burnetii* have been found in this soft tick species (Pader et al. 2012; Lafri et al. 2017; Boucheikhchoukh et al. 2018; Hosseini-Chegeni and Kayedi 2020). Only recently in Pakistan, it was reported that *A. persicus* collected from domestic poultry harboured DNA of *Toxoplasma gondii* (Khan et al. 2024). However, it is unclear the role of this tick in the transmission of these pathogens and only transmission studies will provide some clarity in this regard. *Dermanyssus gallinae* and *Knemidocoptes mutans* are other parasitic acarines that was observed in poultry across Nigeria. These two notorious pests occurred at varying prevalences in domestic chickens, turkey and guinea fowl. We observed that in Nigeria, no attempts were made to screen these acarines for pathogens. However, *D. gallinae* has been shown to harbour DNA of several poultry pathogens including *Escherichia coli*, wild-type Marek’s disease virus, wild-type fowlpox virus, fowl adenovirus, chicken anemia virus and fowl adenovirus (Oh et al. 2020). The prevalence of *E. gallinacea* was mainly low to moderate except for one study where a high prevalence of 69.37% was recorded. The distribution of this ectoparasite across the country highlights its significance as an economic important pest in poultry production. The differences in infestation rate have been attributed to various factors such as poor hygiene, lack of treatment of infected chickens and seasonal fluctuations (López-Pérez et al. 2018; Gharsan and Elhassan 2019).

In chickens and ducks across Nigeria, cestodes belonging to the genus *Raillietina* was the most common with *R. echinobothrida*,* R. tetragona*, and *R. cesticillus* as the predominant species. This concurs with observations made in previous other studies conducted in Thailand (Panich and Chontananarth 2021), Bangladesh (Begum et al. 2019), Ethiopia (Eshetu et al. 2001) and Zimbabwe (Mukaratirwa and Hove 2009). These cestode species are highly pathogenic parasites that can cause severe clinical signs, such as epithelial cell inflammation and destruction, nutritional loss, weight loss, slow growth rate, and reduced egg production (McDougald 2020; Chen and Li 2014; Bashini et al. 2017). *Ascaridia galli* is regarded as the largest nematode of chickens and the most frequently reported endoparasites of free-range chickens. It is one of the most widely prevalent and distributed parasites of free-range birds. Their prevalence varied widely across several regions in Nigeria with up to 86.0% occurrence. The variation in the prevalence rate of ascaridiosis in different studies may be due to difference in the geographical location of the research areas, sample size, age of the birds, use of dewormer and methods of detection (AbouLaila et al. 2025). In Bangladesh, the decreasing trends of the prevalence of the worm may be due to the increased access of the veterinary services, awareness of the people and the gradual motivation of people regarding harmful effects of the helminths as well as the positive changes of their attitude and practice of regular deworming (Ritu et al. 2024). In Nigeria, *A. galli* have been detected in chickens, guinea fowl, Muscovy duck, pigeon, quails, turkey and weaver (Ogbaje et al. 2012; Opara et al. 2014; Ola-Fadunsin et al. 2019b; Yahaya et al. 2022; Onyeabor and Uwalaka 2021; Ombugadu et al. 2021). Affected birds with this parasite were emaciated, anaemic with reduction in production efficiency (Dänicke et al. 2009; Phiri et al. 2007). Consequently, infection reduce meat and egg production with intestinal haemorrhage when they is high parasite burden (Shifaw et al. 2023). Among gastrointestinal nematode, *Heterakis gallinarum* was the second most predominant and widespread. This cecal nematode of poultry is generally found wherever poultry are reared (Madsen 1950). Infection with this parasite is responsible for reduced weight gain in cases of heavy infection as observed in other ascarid infections (Ikeme 1971; Kaushik and Deorani 1969). The prevalence varies considerably across the North and South of Nigeria with one study recording a high prevalence of 79.0% in Gombe, Northeastern Nigeria. No particular reason could be attributed to this exceptional high prevalence. However, it has been postulated that the prevalence is influenced by several factors including farm management parameters such as stocking density, feeding and nutrition and climate (Kaufmann et al. 2011; Thapa et al. 2015). Gallinaceous birds including chicken, Muscovy duck, turkey, guinea fowl, geese, quail and water fowl were documented in Nigeria to be infected by this worm. Our observation agrees with previous report where it was shown that several avian hosts are known to be infected by this nematode (Cupo and Beckstead 2019). However, the majority of the reports in Nigeria was in the domestic fowl. This reiterates the importance of chickens as carriers for the nematode and prominent reservoirs for *Histomonas meleagridis*. Furthermore, the high prevalence of gastrointestinal helminth infections recorded in poultry, particularly among free ranging chickens could be due to their free-ranging habit which allows them free access to infective stages of parasites through coincidental ingestion of arthropods like beetles, earthworms, ants which act as intermediate hosts to most of the helminth parasites while in search of feed (Soulsby 1982). Infection of poultry with trematodes were few with only a single report. This observation could be due to less availability of wet habitats for chicken to ingest molluscs that act as the first-intermediate hosts for the hatched trematode larvae to reproduce asexually to generate free-swimming cercariae (Mathews et al. 2024).

Avian haemasporidian are vector-borne parasites of the genera *Plasmodium*, *Haemoproteus*, and *Leucocytozoon* and are known to infect blood cells of diverse avian host across all zoogeographical regions (Valkiunas 2005). Among the three haemoparasites, infection with *Plasmodium* species was the most predominant and widespread. *Plasmodium* spp. causes avian malaria in poultry resulting in progressive anaemia, emaciation, and enlargement of spleen and liver in affected birds (Soulsby 1982). In commercial poultry production, avian malaria is not of major veterinary importance. However, among free ranging birds, it can result in high mortality rates (Opara et al. 2014). *Haemoproteus* infection is not particularly pathogenic in domestic chicken (Soulsby 1982), however, domestic birds both in the north and southern regions of Nigeria were documented to be infected by *Haemoproteus* spp. Infection of poultry with *Leucocytozoon* spp. was the least documented and this could be attributed to agro climatic variation which affects vector distribution and adaptation of the *Leucocytozoon* spp., in different agro climatic zones. *Leucocytozoon* spp infection causes anaemia, thickened oral discharge and paralysis of legs (Sadiq et al. 2003). Furthermore, the variation in prevalence of haemoparasites in avian species may be attributed to differences in the host susceptibility, availability of competent vectors, differences in species or strain of the vectors and climatic conditions which affect the distribution and spread of vectors, and the avian health management programme (Richard et al. 2002; Szöllősi et al. 2011; Atkinson et al. 2014; Vaisusuk et al. 2022). The vast majority of studies employed the classical microscopic examination of blood film only to detect haemoparasitic infection. This made it difficult for the taxonomic classification of the various blood parasites to the species level due to lower resolution. Nonetheless, microscopy is regarded as the gold standard to diagnose *Plasmodium* infection and other haemoparsites in both humans and birds (Permin 1998; Okanga et al. 2013).

## Limitations of the study

This review was basically a narrative review and not ‘systematic and meta-analysis’. The data presented on the tables was limited to surveys carried out in Nigeria that were published in peer-reviewed journals. In each geopolitical zone, not all states have carried out survey to investigate the occurrence of gastrointestinal parasites and the reports from that region may not be a true representation of the situation for that zone. We hope that future studies will attempt to carry out a systematic review and meta-analysis and present the outcome of the summarize data in a more appropriate manner to decipher the current occurrence of endo-, ecto and haemoparasites of poultry across Nigeria.

## Concluding remarks and final perspective

This review has compiled literature using available evidence from published journals articles on avian parasites in Nigeria with particular emphasis on ecto-, endo- and haemoparasites that afflicts them. Endo-parasites of avian classified into three major groups were registered to afflict poultry in Nigeria in the last 40 years. From the available evidence, it was obvious several species of nematodes have been registered and they include *Ascaridia galli*,* Heterakis gallinarum*,* Subuluru brumpti* and *Capillaria* species. With regards to cestodes, some of the documented species from published literature were *Raillietina* species (specifically *R. tetragona*,* R. cesticillus* and *R. echinobothrida*) and *Choanotaenia infundibulum*. *Prosthogonimus species* was the only documented trematodes infecting poultry in Nigeria. On the side of ecto-parasites, almost all the major groups that includes ticks (*Argas persicus*,* Ar. walkerae* and *Ornothodoros moubata*), mites (*Dermanyssus gallinae* and *Knemidocoptes mutans*), fleas (*Echinophaga gallinacean*) and lice (*Menacanthus stramineus*,* Menopon gallinae*, *Lipeurus caponis* and *Columbicola columbae* ) have all been registered on poultry across the country. Lastly, protozoan parasites including haemoparasites (*Plasmodium* spp., *Haemoproteus* spp. and *Leucocytozoon* spp.) and coccidian (*Eimeria* spp. and *Cryptosporidium* spp.) were observed to infect poultry in Nigeria. All these parasites enlisted above were recorded on different poultry types with varying prevalence’s across the six geopolitical zones of Nigeria. Despite the recovery and identification of several vectors and arthropod types, no singular report exists in Nigeria that attempt to screen the collected vectors for vector-borne pathogen. This underscores the low penetration level of molecular based diagnostic technique in Nigeria. It is obvious from this study that the application of molecular diagnostics in the characterization of poultry parasites have low penetration in Nigeria. Over 95.0% of published studies relied solely on the classical parasitology technique of microscopy for the detection of helminth ova, identification of arthropod and examination of thin blood film for haemoparasites. Consequently, this has limited the identification of some of these parasites to the genus level as against the species level which can be difficult with the microscopic technique most especially for haemoparasites when the parasitaemia is low. Furthermore, we also observed that in some states in Nigeria, they is paucity of studies or complete absence on avian parasites. We are advocating that the government make available research grant that focuses on studies that investigates poultry diseases so that research interest can be stimulated in this direction. Additionally, knowledge gap can be enhanced through research collaboration among academics and research and this paper is a clarion call for avian medicine specialist and parasitologist to rise to the occasion. Lastly, large scale surveys covering more extensive geographical areas and poultry species are necessary in order to provide a broader understanding of the impact of poultry endo-, ecto and blood-borne parasites in Nigeria. These intensive surveys are required to determine the spatial and temporal distribution, type, significance and economic importance of poultry parasites that will contribute to the development of technically feasible and economically viable control strategies rather than depending on poultry keeper’s efforts only.
